# Complications of clozapine cessation in the context of chemotherapy-related neutropenia—a case report

**DOI:** 10.3389/fonc.2026.1863699

**Published:** 2026-10-06

**Authors:** Colm Sweeney, Gavin Rush, Sarah O’Dwyer, Colm Mac Eochagain, Róisín Plunkett

**Affiliations:** 1 Department of Psychological Medicine, St James Hospital, Dublin, Ireland; 2 St Patrick’s University Hospital, Dublin, Ireland; 3 Department of Oncology, St James Hospital, Dublin, Ireland

**Keywords:** breast cancer, catatonia, chemotherapy, clozapine, neutropenia

## Abstract

**Background:**

Clozapine is the gold standard for treatment-resistant schizophrenia (TRS). It requires strict monitoring of full blood count (FBC), due to the risk of neutropenia and agranulocytosis.

**Case history:**

AB is a woman in her mid-40s with TRS, diagnosed with invasive ductal carcinoma of the right breast. She was commenced on a chemotherapy regimen of docetaxel, carboplatin, trastuzumab, and pertuzumab. Four days after the initial infusion, AB developed neutropenia with an absolute neutrophil count of 0.2 × 10^9^/L. In accordance with local protocols and discussion with the Clozapine Patient Monitoring Service (CPMS), clozapine was immediately discontinued. Two weeks after clozapine cessation, AB displayed catatonic symptoms characterised by mutism, posturing, and automatic obedience, scoring 27 on the Bush–Francis Catatonia Rating Scale (BFCRS). A positive lorazepam challenge confirmed the diagnosis of clozapine-withdrawal catatonia. Despite regular high-dose benzodiazepines and treatment with antipsychotics, AB’s presentation continued to deteriorate, characterised by prominent delusions and hallucinations, impeding oncological treatment. AB was seen extensively by liaison psychiatry and treated with high-dose typical and atypical antipsychotics; however, she failed to respond adequately to treatment. Given AB’s extensive previous history of management on clozapine without previous haematological disturbance and ongoing decline in mental state, coupled with the timeline of symptoms 4 days after the administration of chemotherapy agents associated with neutropenia, namely, carboplatin, the decision was taken to reinstate clozapine. Following titration of clozapine and transfer to a specialist psychiatric unit, AB’s symptoms resolved rapidly, allowing for the successful completion of chemotherapy. Carboplatin was omitted in subsequent cycles, with no further episodes of neutropenia.

**Discussion:**

This case highlights the diagnostic challenge of neutropenia in psychiatric patients on clozapine, who are also undergoing treatment for comorbid medical conditions. The presence of neutropenia in clozapine patients may lead to diagnostic overshadowing and delay the diagnosis of the underlying cause of neutropenia, resulting in delays to treatment as well as unnecessary cessation of clozapine and risk of relapse. Furthermore, this case highlights the risk of catatonia associated with the withdrawal of clozapine and the importance of its rapid identification and management.

**Conclusions:**

Clinicians should consider differing aetiologies of neutropenia in clozapine patients, especially those undergoing chemotherapy. Furthermore, clinicians should be skilled in recognising catatonia and initiating prompt treatment.

## Introduction

Clozapine is an antipsychotic with action at dopamine, 5HT_2A_, and muscarinic acetylcholine receptors (1), among others (2). Clozapine is considered the gold standard for the treatment of treatment-resistant schizophrenia (TRS) (3). It has been shown to be more effective than other antipsychotic agents in the reduction of symptoms in patients with TRS (4).

Catatonia is a severe neuropsychiatric syndrome which affects emotion, speech, movement, and behaviour (5). Catatonia can present in varied forms, with increased or decreased activity, as well as with purposeless movements (6). Catatonia is often associated with severe psychiatric illness; however, it has myriad aetiologies including underlying medical illness (7).

The onset of catatonia has also been associated with the withdrawal of clozapine in patients previously stabilised on the medication (8, 9).

The concurrent administration of clozapine and anti-cancer drugs presents management challenges for clinicians due to potential drug interactions including changes in clozapine serum concentration, QTc prolongation, myelosuppressive effects, sedation, and constipation (10).

We present an unusual case of catatonia in the context of abrupt cessation of clozapine in a patient receiving chemotherapy for breast cancer.

## Patient information (medical, family, and psychosocial history)

AB is a woman in her mid-40s diagnosed with invasive ductal carcinoma (IDC) of the right breast in June 2025, which was oestrogen receptor (ER) and HER2 receptor positive on biopsy.

AB has a pre-existing diagnosis of treatment-resistant schizophrenia and was managed on clozapine 300mg once daily. She had been well managed for over 10 years and had not experienced any psychotic relapses during her treatment with clozapine. AB had been compliant with regular full blood count (FBC) monitoring for clozapine. Her white cell count (WCC) completed in the month before presentation, July 2025, was within the normal range of clozapine monitoring parameters: WCC greater than 3.5 × 10^9^/L and an absolute neutrophil count (ANC) greater than 2 × 10^9^/L (11). AB had no history of receiving a ‘red result’ on FBC monitoring, confirming that she had never had an ANC of less than 1.5 × 10^9^/L or a WCC less than 3 × 10^9^/L during her treatment with clozapine (11).

AB had been diagnosed with autism spectrum disorder (ASD) prior to her cancer diagnosis following an assessment recommended by her treating psychiatry service. The treating psychiatry service had considered it possible that the diagnosis of schizophrenia could be due to misinterpretation of features of ASD, but had not yet trialled reduction of clozapine. AB had previously been treated for a diagnosis of bipolar affective disorder (BPAD) when previously attending a private clinic; however, there was some diagnostic and medication uncertainty in relation to her treatment, and when her care was transferred to her current service, the diagnosis was revised to one of schizophrenia.

AB is single with no children, works as a civil servant, and has good relationships within her family and community. She has a family history of prostate cancer, bowel cancer, renal cancer, and head and neck cancer on her paternal side.

## Timeline

### Clinical findings

Following IDC diagnosis, AB commenced on a first cycle of docetaxel, carboplatin, trastuzumab, and pertuzumab (TCHP) in August 2025. Four days after this, AB developed chest pain and dyspnoea. She attended the emergency department (ED) of her local hospital and was admitted there. Examination showed pyrexia of 38.2 °C, and investigations revealed leukopenia of 0.6 × 10^9^/L and a grade 4 neutropenia with an ANC of 0.2 × 10^9^/L.

Given the neutropenia, the liaison psychiatry service in the local hospital service discussed the case with the Clozaril Patient Monitoring Service (CPMS).^1^ CPMS advised that, given the episode of neutropenia, clozapine should be stopped immediately.

Clinical notes indicated that neutrophils rose back to within the normal range at 5.1 × 10^9^/L 4 days after presentation to the hospital and cessation of clozapine and 8 days after the administration of the first cycle of TCHP. There were no further episodes of neutropenia during the inpatient treatment course.

In the first week of admission to her local hospital, AB was seen regularly by a liaison psychiatry team. She was noted to be anxious in presentation and treated with escitalopram, a selective serotonin reuptake inhibitor (SSRI), and quetiapine, an atypical antipsychotic, as well as diazepam 2 mg *nocte* (**Figure 1**).

**Figure 1:**
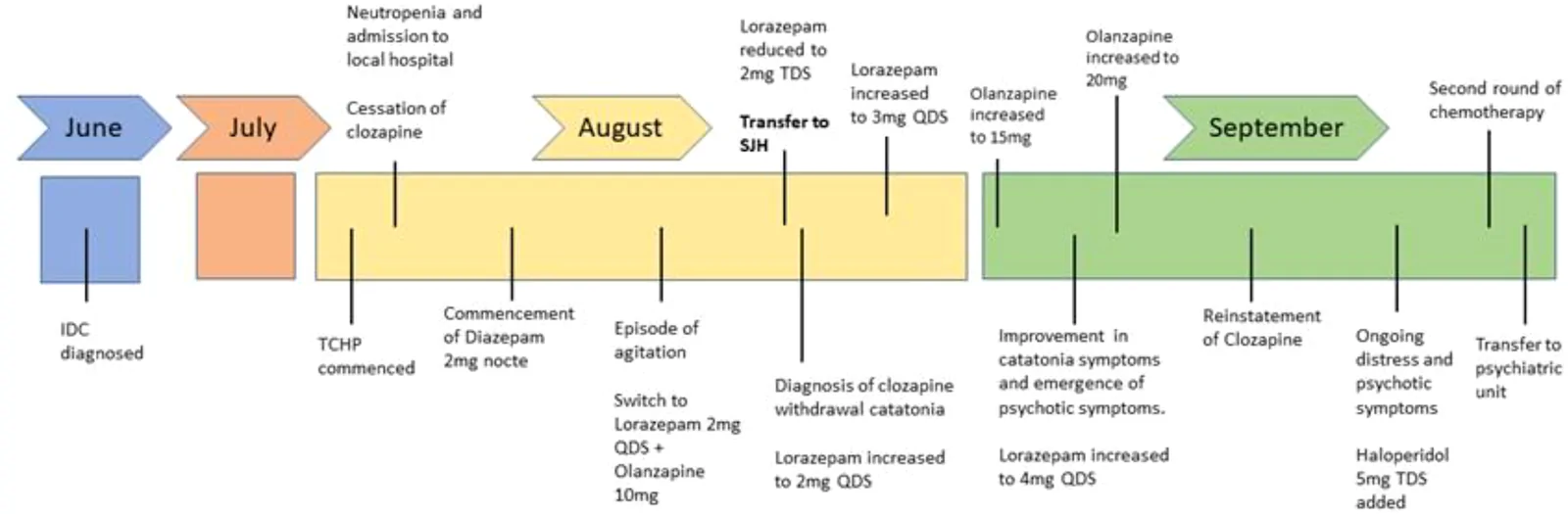
Timeline of the case presentation and treatment.

One week into that admission, AB was noted to develop vague paranoia and suspiciousness with associated mild pressure of speech. Her presentation at this time was also notable for an episode of agitation and aggression, in which she reportedly shouted at staff and smashed a glass on the floor, requiring rapid tranquilisation with lorazepam to manage her acute distress. The presumptive diagnosis at that time was delirium.

At this point, medications were switched to olanzapine 10 mg *nocte* and lorazepam 2 mg QDS. Documentation suggests that AB responded well to the change of treatment because there were no further episodes of aggression or agitation noted. However, there were ongoing episodes of intermittent confusion and paranoid symptoms documented. For example, on day 14 of her admission, she reportedly stated that the devil was in her room with her.

Later that day, she was transferred to a tertiary centre, a designated OECI Comprehensive Cancer Care centre, under the care of the consultant oncologist who had been treating her (**Figure 2**).

**Figure 2:**
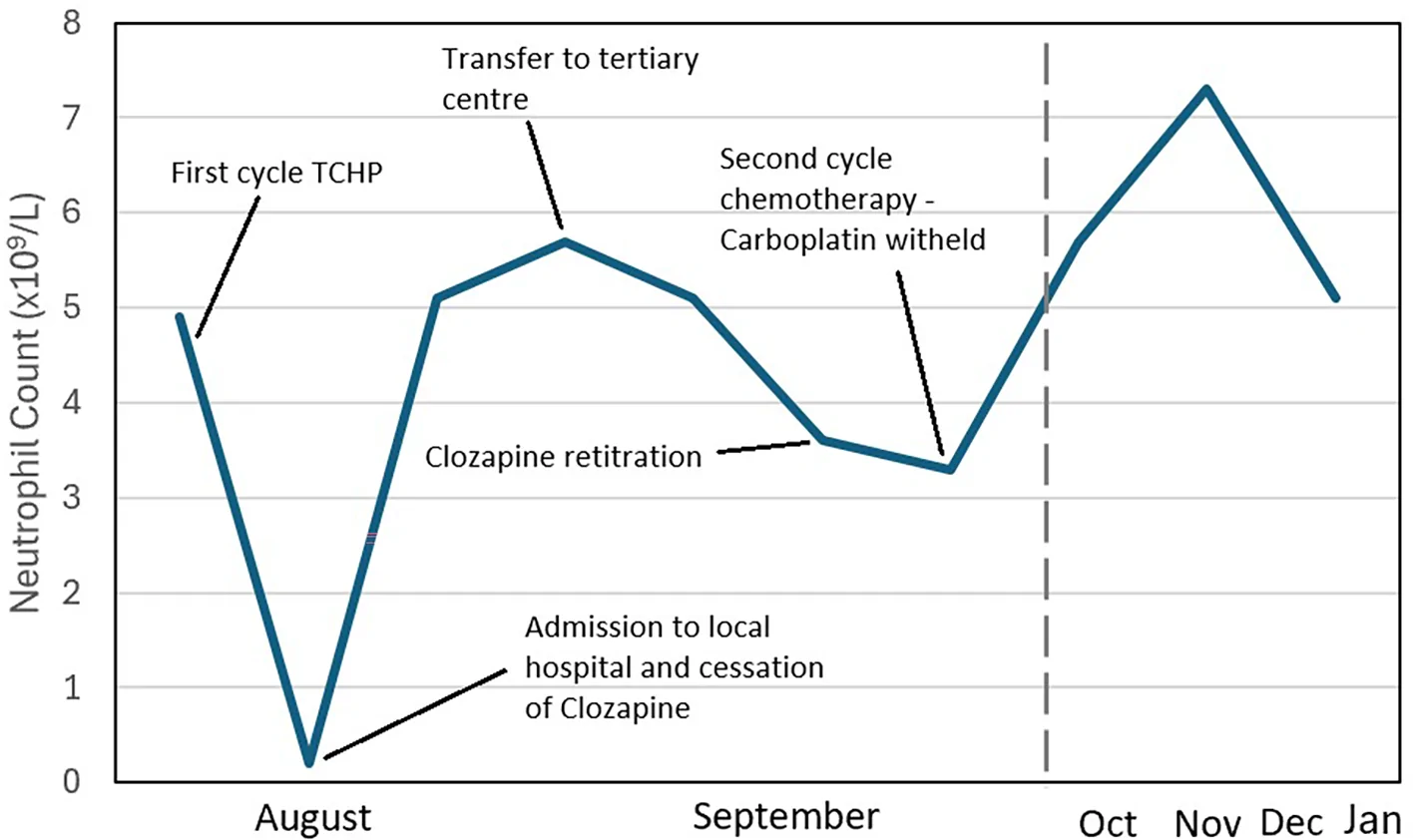
Neutrophil count during the treatment course.

On transfer, her medications were as follows: clozapine 300 mg OD (on hold), pantoprazole 40 mg OD PO, promethazine 25 mg OD PO *nocte*, escitalopram 10 mg OD PO *mane*, lorazepam 2 mg TDS PO, and olanzapine 10 mg OD PO *nocte*.

### Diagnostic assessment

On admission to the tertiary centre, notes from the admitting doctor at this time indicate that AB was unable to engage with the admission procedure on arrival. She did not answer any questions asked in order to complete admission history. Of note, AB refused night-time medications, so she did not receive the *nocte* dose of olanzapine or lorazepam.

AB was reviewed by the psychological medicine team (psychiatry and nursing) the following morning. On review, she was uncommunicative and was unable to engage meaningfully in conversation. Clinical examination demonstrated signs of catatonia including mutism, posturing, stereotypy, ambitendency, and automatic obedience.

AB was assessed for catatonia by the liaison psychiatry team with a score of 27 on the Bush–Francis Catatonia Rating Scale (BFCRS).^2^

The working diagnosis at that time was catatonia secondary to withdrawal of clozapine 2 weeks prior. Lorazepam challenge of 1 mg intravenously (IV) was administered. Two hours after administration of IV lorazepam, the BFCRS score was 12 (a reduction of 15 points from initial assessment, denoting a positive lorazepam challenge). Based on response to lorazepam challenge, regular lorazepam 2 mg IV QDS was commenced.

Over the following days, AB was noted to make gradual improvements with more goal-directed activity observed; however, she remained non-communicative verbally. She required assistance with all ADLs and was not eating or drinking spontaneously.

Lorazepam was increased to 3 mg QDS on day 4 of admission. Olanzapine was increased to 15 mg *nocte*. Promethazine was stopped on day 7.

Based on the clinical picture, the oncology service postponed the second cycle of TCHP and decided to withhold carboplatin from the next cycle to minimise the possibility of further episodes of neutropenia, given its previously documented association with neutropenia (15).

On days 7–12 of admission, AB was noted to be more spontaneous in speech and interaction, indicating an improvement in catatonia symptoms. However, the content of her speech and writing was concerning, with references to the devil and to her family being in danger.

On day 14 of admission, AB reported clear persecutory beliefs that her parents were in danger and that she was going to be sent to hell. Catatonia was noted to be improving at this time, with psychomotor slowing and abnormal movements largely resolved, though oral intake remained low. Lorazepam was increased to 4 mg QDS and haloperidol was added on an as-required (PRN) basis.

Over the following days, the patient continued to express delusional beliefs including that her parents had been replaced by imposters, that staff on the ward were clones, and that she was able to communicate with Satan. Lorazepam was switched from IV to oral formulation, remaining at 4 mg QDS on day 18 of admission. Olanzapine was further increased to 20 mg on day 21.

Unfortunately, despite the increase in olanzapine, AB continued to experience a relapse of psychosis with associated distress and agitation.

### Therapeutic intervention

It was therefore clear that further treatment with olanzapine was unlikely to be of benefit and that reinstatement of clozapine was required. Following a discussion and agreement with AB’s usual treating psychiatrist, contact was made with CPMS in order to request off-licence prescribing of clozapine. In view of the severe ongoing distress and psychotic symptoms, previous stability on clozapine, and the strong likelihood of neutropenia being secondary to carboplatin, rather than clozapine, it was agreed that the benefits of reinstating Clozapine outweighed the risks of doing so.

Clozapine was restarted on day 25 at a dose of 12.5 mg with a daily titration schedule, while lorazepam was reduced to 14 mg total dose per day.

AB continued to experience significant distress and agitation necessitating the use of PRN medication. On day 26, she continued to experience significant agitation requiring the use of haloperidol 20 mg in 24 h, in addition to the regularly prescribed clozapine 25 mg, olanzapine 20 mg, and lorazepam 14 mg. Haloperidol was changed to a regular dose of 5 mg TDS at this point, with good effect noted on agitation and distress; however, AB continued to experience persecutory beliefs.

On day 28 of admission, in light of escalating agitation and psychotic symptoms, it was felt that continued treatment in a general hospital setting was becoming untenable, and the best course of action was to facilitate transfer to a specialised psychiatric unit for further management.

The oncology team advised that AB was due to receive a further cycle of chemotherapy the following day, and it was agreed to facilitate its administration while she remained an inpatient and then seek transfer following this.

Following administration of chemotherapy, a discussion was conducted with AB and her family about transfer to a psychiatric unit for more specialised treatment. AB was not agreeable to this, reporting that she believed that her life and her family’s safety would be under threat if she were transferred to the psychiatric unit. Given AB’s lack of insight, the need for treatment, and the risks of not administering treatment in a specialist inpatient setting, application and recommendation for transfer to a specialist psychiatric unit under the Mental Health Act 2001 were completed. This transfer occurred on day 30 of admission to a tertiary centre. Clozapine dose was rapidly titrated to 300 mg, with swift resolution of psychotic symptoms.

### Follow-up and outcomes

This rapid response allowed for her transfer back to the tertiary service 3 weeks later for continuation of chemotherapy. AB was reviewed by the Psychological Medicine Service on readmission and was well, with no features of catatonia or active psychosis. She displayed good insight into her condition. Carboplatin was administered for future cycles.

AB remained well throughout the treatment, completing scheduled chemotherapy and later undergoing mastectomy under a breast surgery team without complications. There were no further episodes of neutropenia during treatment or post-chemotherapy while receiving clozapine.

## Discussion

This case highlights the potential for clozapine use to overshadow other potential causes of neutropenia in patients with treatment-resistant schizophrenia and the potential harm caused by abrupt cessation of clozapine.

While clozapine can be associated with neutropenia and agranulocytosis (1, 16), in this case, there is no evidence to suggest that neutropenia was caused by clozapine. Chemotherapy-induced neutropenia (CIN) is a recognised consequence of chemotherapy, with all patients undergoing chemotherapy having a risk of developing CIN (17). The risk of developing CIN is highest after the first cycle of chemotherapy, compared with subsequent cycles (18).

As regards the TCHP chemotherapy regimen, there are differing timelines and mechanisms in relation to ANC nadirs typically seen with each agent. Docetaxel, a chemotherapeutic agent belonging to the taxane class, promotes cell microtubule stabilisation, leading to impairment of cell mitosis and arrest of the cell cycle (19), and has a median ANC nadir time of 7 days from initiation (20). Carboplatin is associated with a time to nadir myelosuppression of 21 days (21) and acts as an atypical alkylator, cross-linking DNA and leading to strand breakage during replication (22). Pertuzumab is a humanised monoclonal antibody that exerts antitumour effects via antibody-dependent, cell-mediated cytotoxicity (23). The addition of pertuzumab to existing treatment combinations has been associated with increased rates of febrile neutropenia, compared with controls (23).

Growing research has been published documenting clinical challenges of concurrent administration of clozapine and chemotherapy with respect to neutropenia and continuation of clozapine. A number of reviews of similar case reports describe relapse of psychotic symptoms after clozapine cessation and subsequent successful reintroduction of the medication (2, 24, 25). Systematic reviews of the literature demonstrate that the risk of clozapine-associated neutropenia is highest in the initial period of treatment, with greatly decreased rates in those on long-term stabilised treatment (2), underlining that clozapine may not be the causative agent of neutropenia in cases of recent initiation of concurrent chemotherapy. Previously documented reviews of the literature argue that clozapine is a feasible option in those who are already stabilised on the medication and are facing the prospect of starting chemotherapy (26).

Prescribers and patients should be aware of this, and off-licence prescribing arrangements for clozapine should be initiated where appropriate.

It should be noted that the ANC nadir occurred prior to the typically seen timeframes of the anti-cancer agents used, suggesting that an interplay between the agents used and clozapine may be a potential explanation for the rapid drop in neutrophils seen in this case.

Second, this case underscores the importance of prompt recognition of catatonia and its causes, including withdrawal of clozapine and other medications. Clinicians should be aware of the signs of catatonia and should have a high index of clinical suspicion for the syndrome in patients presenting with changes in movement, speech, emotion, or behaviour. Clinicians should familiarise themselves with using a standardised rating scale for catatonia, such as the BFCRS, as it provides diagnostic confidence and is an objective method of monitoring response to treatment.

Identification of catatonia occurred on the day of transfer to the tertiary centre and allowed for prompt initiation of appropriate and effective treatment. It is possible that the lower dose of lorazepam prescribed in the local hospital for anxiety partially treated the catatonic symptoms. Having missed the doses of lorazepam during transfer from the local hospital, the patient developed significant catatonic symptoms on day 14 of admission. However, literature indicates that the onset of clozapine withdrawal catatonia can occur within a timeline of approximately 48 h to 2 weeks (26). It is therefore unclear whether, in this case, catatonia was absent, partially treated, or present but not identified before transfer. It may be that onset of catatonia on transfer was coincidental and unrelated to previous adjustment of benzodiazepines. Recent evidence suggests that, where possible, clozapine should be downtitrated over a period of 6 months to minimise potential for withdrawal symptoms (27).

Third, this case highlights the complexity of mental state assessment in the case of comorbidity. AB’s diagnosis of ASD and long period of stability since commencement of clozapine had led to uncertainty around the veracity of the diagnosis of treatment-resistant schizophrenia. That, in combination with the impact of catatonia on AB’s mental state, led to a diagnostic dilemma. Without the comorbidity and diagnostic uncertainty, reinstatement of clozapine would likely have been undertaken earlier in the clinical course.

Finally, this vignette demonstrates the importance of psychological medicine/liaison psychiatry input for treating patients with psychotic symptoms in oncology settings. Due to close collaboration between psychiatry and oncology services, in this case, oncological treatment and effective treatment of catatonia and psychosis were possible simultaneously in the acute phase. Liaison with specialist inpatient psychiatric services then allowed appropriate ongoing care. This facilitated continued adherence to chemotherapy and appropriate reinstatement of clozapine.

## Conclusions

Clinicians should consider all the potential aetiologies of neutropenia in patients prescribed clozapine, especially those undergoing chemotherapy. Clinicians should be skilled in recognising catatonia and initiating prompt treatment. Psycho-oncology services should have dedicated psychiatry resourcing to allow for appropriate management of complex cases.

## Data Availability

The original contributions presented in the study are included in the article/supplementary material. Further inquiries can be directed to the corresponding author.

## Footnotes

1 CPMS is an international haematological monitoring service for WCC results in clozapine patients in the UK and Ireland, with which all clozapine patients must be registered prior to commencing clozapine (12)

2 The Bush–Francis Catatonia Rating Scale (BFCRS) is a rating tool that includes 23 clinical findings of catatonia, and is the most commonly used scale to identify and measure catatonia in clinical settings (13). The presence of two or more of the first 14 criteria indicates a positive result, while the subsequent nine criteria are used to measure severity (13). Each criteria is measured on a 0–3 point scale, giving a possible score range of 0–69 (14).
